# Mid-term outcomes of the Absorb BVS versus second-generation DES: A systematic review and meta-analysis

**DOI:** 10.1371/journal.pone.0197119

**Published:** 2018-05-09

**Authors:** Cordula M. Felix, Victor J. van den Berg, Sanne E. Hoeks, Jiang Ming Fam, Mattie Lenzen, Eric Boersma, Peter C. Smits, Patrick W. Serruys, Yoshinobu Onuma, Robert Jan M. van Geuns

**Affiliations:** 1 Thorax centre, Erasmus Medical Centre, Rotterdam, the Netherlands; 2 Cardiology department, National Heart Centre Singapore, Singapore; 3 Cardiology department, Maasstad Hospital, Rotterdam, the Netherlands; 4 Cardiology department, The National Heart and Lung Institute, Imperial College London, London, United Kingdom; 5 Cardiology department, Radboud UMC, Nijmegen, the Netherlands; Universita degli Studi Magna Graecia di Catanzaro, ITALY

## Abstract

**Background:**

Bioresorbable Vascular Scaffolds (BVS) were introduced to overcome some of the limitations of drug-eluting stent (DES) for PCI. Data regarding the clinical outcomes of the BVS versus DES beyond 2 years are emerging.

**Objective:**

To study mid-term outcomes.

**Methods:**

We searched online databases (PubMed/Medline, Embase, CENTRAL), several websites, meeting presentations and scientific session abstracts until August 8^th^, 2017 for studies comparing Absorb BVS with second-generation DES. The primary outcome was target lesion failure (TLF). Secondary outcomes were all-cause mortality, myocardial infarction, target lesion revascularization (TLR) and definite/probable device thrombosis. Odds ratios (ORs) with 95% confidence intervals (CIs) were derived using a random effects model.

**Results:**

Ten studies, seven randomized controlled trials and three propensity-matched observational studies, with a total of 7320 patients (BVS n = 4007; DES n = 3313) and a median follow-up duration of 30.5 months, were included. Risk of TLF was increased for BVS-treated patients (OR 1.34 [95% CI: 1.12–1.60], p = 0.001, I^2^ = 0%). This was also the case for all myocardial infarction (1.58 [95% CI: 1.27–1.96], p<0.001, I^2^ = 0%), TLR (1.48 [95% CI: 1.19–1.85], p<0.001, I^2^ = 0%) and definite/probable device thrombosis (of 2.82 (95% CI: 1.86–3.89], p<0.001 and I^2^ = 40.3%). This did not result in a difference in all-cause mortality (0.78 [95% CI: 0.58–1.04], p = 0.09, I^2^ = 0%). OR for very late (>1 year) device thrombosis was 6.10 [95% CI: 1.40–26.65], p = 0.02).

**Conclusion:**

At mid-term follow-up, BVS was associated with an increased risk of TLF, MI, TLR and definite/probable device thrombosis, but this did not result in an increased risk of all-cause mortality.

## Introduction

Bioresorbable scaffolds, developed to overcome some of the (late) adverse events of metallic drug-eluting stents (DES), are the latest innovation in the treatment of coronary artery disease. The Absorb bioresorbable vascular scaffold (BVS, Abbott Vascular, Santa Clara, CA, USA) is the most intensively studied. The first-in-man study in 2006 revealed promising results and this new device received a CE-mark in 2011 and became commercially available in Europe in September 2012. FDA approval followed in 2016 [1].

The concept of the Absorb BVS consists of treatment of obstructive coronary artery disease with temporary support of the vessel wall while avoiding the acute complications of balloon angioplasty. It was hypothesized that complete resorption would result in restoration of vasomotion, a reduction in angina, and the avoidance of caging of the vessels or interference with non-invasive imaging. In addition, vessel geometry would be less affected after implantation of a BVS. This should result in better outcomes for patients, with reduced late event rates. Pooled individual data from the four largest randomized controlled trials (RCTs) comparing BVS with second-generation DES did support the concept of temporary support of the artery and showed non-inferiority of the device during the first year [2]. However, several meta-analyses that included data beyond 1 year revealed higher event rates of myocardial infarction, target lesion revascularization and scaffold thrombosis [3, 4]. Data on the performance of BVS beyond 1 year primarily came from small registries, propensity-matched observational studies and a few RCTs. These raised concerns about the occurrence of very late (after 1 year) scaffold thrombosis [5], whereas RCTs assessed only the mid-term time points. We therefore undertook this systematic review and meta-analysis, and report the mid-term clinical outcomes of the Absorb BVS compared with second-generation DES.

## Methods

### Data sources and study selection

Inclusion criteria for our study were RCTs comparing the Absorb BVS with the Xience CoCr-EES, a second-generation DES, in patients with coronary artery disease with > 12 months of follow-up available. As randomized mid- to long-term data are scarce, we also allowed propensity-matched observational studies comparing BVS with second-generation DES. Both full-length manuscripts and meeting presentations (containing unpublished data) were included. All studies had to report on the outcomes of interest and be written in English. Exclusion criteria were non-human studies, single-arm studies, imaging-only studies, studies with short follow-up (≤ 12 months), studies in <100 patients, review articles, case series, trial design articles, comparisons other than Absorb BVS versus second-generation DES, studies with duplicate data, and those where the scaffold or stent was implanted elsewhere than in the coronary artery. This meta-analysis was conducted in accordance with the Preferred Reporting Items for Systematic Reviews and Meta-analysis guidelines [6] (S4 Table).

### Data extraction and quality assessment

On August 8^th^, 2017, a medical librarian (WB) conducted a systematic search of the online databases Medline/PubMed, Embase and Cochrane Central Register of Controlled Trials (CENTRAL), several websites (e.g. http://www.clinicaltrials.gov) and scientific session abstracts and oral presentations from conferences, with the following keywords and corresponding MeSH terms: “drug-eluting stent(s)”, “everolimus-eluting stent”, “bioresorbable vascular stent”, “bioresorbable scaffold”. On October 31^th^, during the 2017 TCT congress, ABSORB II, III and TROFI II presented their 3- and 4-year outcomes, which we also included in our analysis. The bibliographic records retrieved were imported and de-duplicated in Endnote bibliographic software. Two physician reviewers (CF and VB) independently screened the records for eligibility at title or abstract level. Records that were relevant were downloaded and full text manuscripts or meeting presentations were reviewed. Differences between reviewers regarding study selection or data extraction were resolved by consensus. If one study had multiple publications with different follow-up lengths, the most recent follow-up record was used.

Quality and risk of bias in reporting data were assessed according to the Cochrane Handbook of Systematic Reviews [7] and by using the Newcastle-Ottawa Quality Assessment scale for case-control studies (maximum score = 9, meaning low risk of bias). Publication bias for the primary endpoint was assessed using funnel plot.

### Outcomes and definitions

The primary outcome for this analysis was target lesion failure (TLF), a composite endpoint that consists of cardiac death, target-vessel myocardial infarction and ischemia-driven TLR. Secondary outcomes were all-cause mortality, all myocardial infarction, ischemia-driven TLR and definite or probable device thrombosis. Deaths were considered cardiac unless a non-cardiac cause was identified. TLR was described as any repeated revascularization of the target lesion. Device thrombosis was classified according to the Academic Research Consortium [8]. To investigate the effect of the intended bioresorption of the device, we examined outcomes during the first and second years separately. Definitions of clinical outcomes per study are described in S1 Table.

### Statistical analysis

Odds ratios (ORs) with 95% confidence intervals (CIs) were used as summary statistics across all studies and were calculated using a random effects model (Dersimonian and Laird). We also provide results of the fixed-effect model. Treatment effect was not assessed in studies in which no events were reported. Heterogeneity was assessed using Cochran Q and Higgins I^2^. I^2^ values of <25%, 25–50% or >50% indicate low, moderate or high heterogeneity. Cochran Q P<0.10 and I^2^>50% were considered to be indicative of significant heterogeneity. All analyses were conducted with Revman software (version 5.3).

Primary and secondary outcomes are reported for all included studies in which the outcome of interest was provided. A sensitivity analysis was performed, as detailed in the online supplement. In this analysis, the treatment effect was investigated in studies that included low-risk patients (ABSORB II, ABSORB III, ABSORB Japan, ABSORB China) versus studies that included more complex population (TROFI II, AIDA, EVERBIO and the observational studies, including higher percentage of STEMI, bifurcation, calcification, long lesions etc.). Finally, separate subgroup analyses for RCTs (low risk of bias) and propensity-matched studies (low/low-moderate risk of bias) were performed.

The risks of adverse events between 0–1 year, 1–2 and 2–3 years were estimated using a landmark population that censored any casualty and lost to follow-up preceding each specific time point.

### Trial sequential analysis

Meta-analyses may results in type 1 errors due to systematic errors (several forms of bias) or random errors (play of chance) due to sparse data and repeated significance testing when a meta-analysis is updated with new trials [9]. This can result in spurious significant results [10]. Trial sequential analysis (TSA) was introduced to minimize random errors. TSA provides the necessary information for meta-analyses and boundaries that determine whether the evidence is reliable and conclusive. We calculated required information size allowing for a type 1 error of 0.05, type 2 error of 0.20, the control event proportions and effect size calculated from the included trials, and heterogeneity estimated by the diversity (D2) in the included trials. We constructed TSA boundaries based on the O’Brien-Fleming alpha-spending function. Trial Sequence Analysis Software (Copenhagen Trial Unit’s TSA Software; Copenhagen, Sweden) was used.

## Results

**Fig 1:**
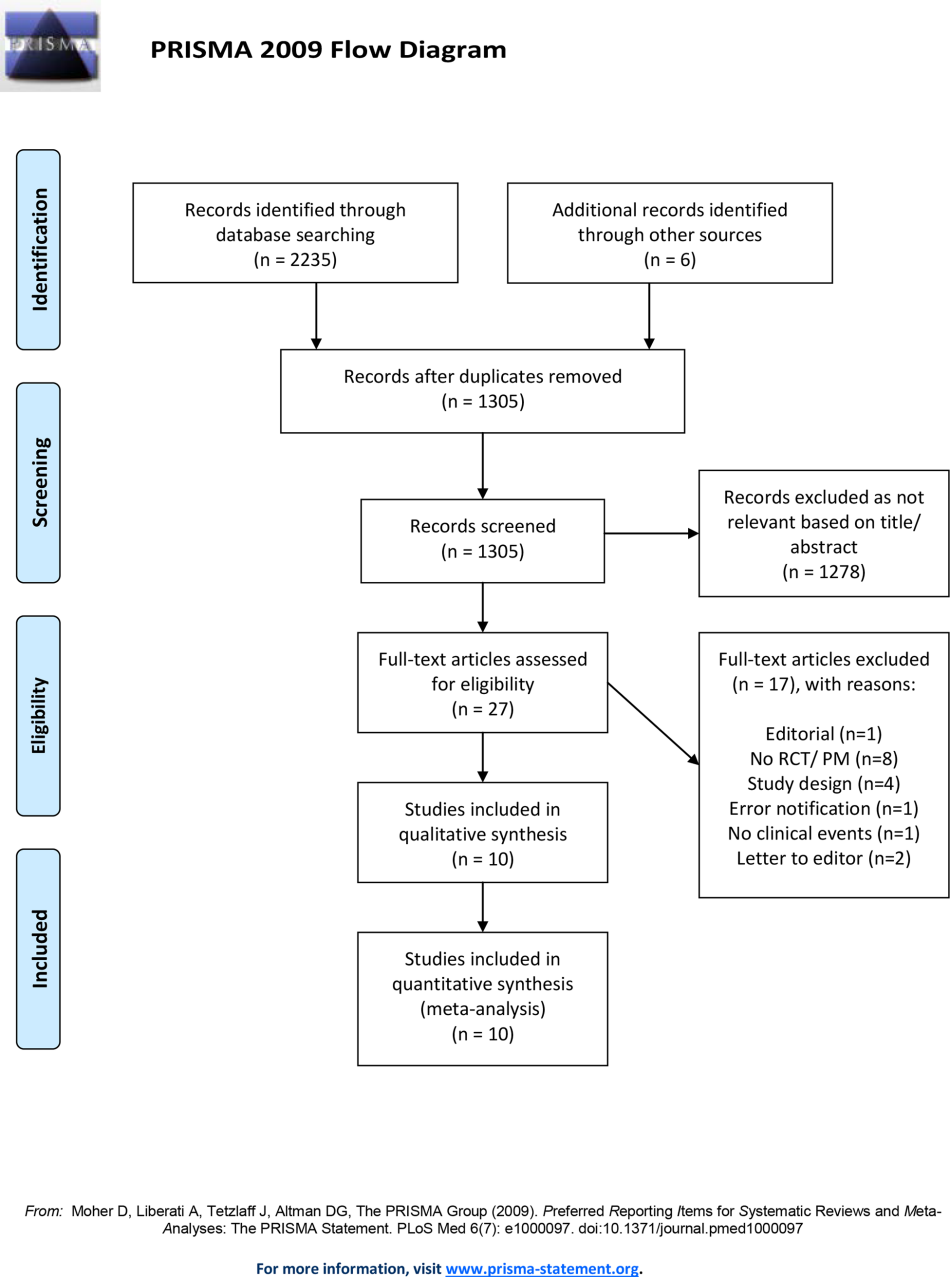
Flowchart.

The de-duplicated results yielded 1305 records. Fig 1 shows a flow diagram of the selection process. Based on the exclusion criteria, 1278 records were excluded after title/abstract review. Twenty-seven records remained for full-text analysis, of which 17 were eliminated (short follow-up or editorials). Ultimately, we included 7 RCTs (3 full-length manuscripts, 4 meeting presentations) with a total of 5578 patients: 3258 received the Absorb BVS and 2320 received a second-generation DES. We also included 3 observational studies (2 manuscripts and 1 meeting presentation) with 1742 patients: 749 were implanted with a BVS and 993 with a DES. Weighted median FU was 30.5 months. Table 1 summarizes the main characteristics of the included studies.

**Table 1:** Major characteristics of included studies.

**Study**	Year	Centres, n	BVS/ DES treated Patients, n	Study type	Clinical presentation	Primary Endpoint	Follow-up, yrs.
**ABSORB II [32]**	2016	46	335/ 166	RCT	SAP, established ACS	Vasomotion & LLL (at 3 yrs.)	1, 2, 3, 4
**ABSORB III [31]**	2017	193	1322/ 686	RCT	SAP, established ACS	TLF (at 1 yr.)	1, 2, 3
**ABSORB Japan [41]**	2016	38	266/ 134	RCT	SAP, established ACS	TLF (at 1 yr.)	1, 2, 3
**ABSORB China [42]**	2016	24	238/ 237	RCT	SAP, established ACS	LLL (at 1 yr.)	1, 2, 3
**TROFI II [30]**	2016	8	95/ 96	RCT	STEMI	HS (at 6 months)	1, 2, 3
**EVERBIO [43]**	2017	1	78/ 80	RCT	SAP, ACS, silent ischemia	LLL (at 9 months)	9 months, 2 yrs.
**AIDA [44]**	2017	5	924/ 921	RCT	SAP, ACS	TVF (at 2 yrs.)	Median of 707 days
**Imori et al. [45]**	2016	8	214/ 215	Propensity matched	ACS	MACE	2
**BVS-Examination [46]**	2016	6	290/ 290	Propensity matched	STEMI	POCE (at 1 yr.)	1, 2
**BVS Expand [47]**	2017	1	244/ 488	Propensity matched	SAP, UA, NSTEMI, silent ischemia	MACE	2

ACS: acute coronary syndrome; DOCE: device oriented composite endpoint; HS: healing score; LLL: late lumen loss; MACE: major adverse cardiac events; RCT: randomized controlled trial; SAP: stable angina pectoris; STEMI: ST-elevation myocardial infarction; TLF: target lesion failure; LLL: late lumen loss; TVF: target vessel failure; UAP: unstable angina pectoris

### Baseline characteristics

Across all studies in this meta-analysis, the mean age of patients ranged from 56.0 to 67.3 years; the percentage of men between 70.1% and 81.4%; diabetic patients between 12.8% and 36.1%; and the percentage of patients that presented with an acute coronary syndrome between 9.8% and 100%. In all studies except ABSORB II and EVERBIO, the per protocol prescribed duration of dual antiplatelet therapy (DAPT) was at least 12 months. The percentage of BVS patients using DAPT at 2 years ranged from 5.5% to 66%. The rate of post-dilatation ranged from 15.2% to 82.2% (Table 2).

**Table 2:** Baseline characteristics (presented as BVS versus EES).

	ABSORB II	ABSORB III	ABSORB Japan	Absorb China	TROFI II	EVERBIO	AIDA	Imori et al.	BVS-Examination	BVS Expand
**Patients**										
**Randomized, n**	355/ 166	1322/ 686	266/ 134	238/ 237	95/ 96	78/ 80	924/ 921	214/ 215	290/ 290	244/ 488
**Age, years**	61.5/ 60.9	63.5/ 63.6	67.1/ 67.3	57.2/ 57.6	59.1/ 58.2	65/ 65	64.3/ 64.0	59.7/ 61.5	56.0/ 57.6	61.3/ 61.9
**Male sex (%)**	76/ 80	70.7/ 70.1	78.9/ 73.9	71.8/ 72.6	76.8/ 87.5	80/ 78	72.5/ 76.0	79.4/ 80.5	81.4/ 79.7	73.4/ 73.6
**Diabetes (%)**	24/ 24	31.5/ 32.7	36.1/ 35.8	25.2/ 23.2	18.9/ 14.7	16/ 22	18.5/ 16.6	14/ 16.7	12.8/ 12.8	18.4/ 20.7
**Hypertension (%)**	69/ 72	84.9/ 85.0	78.2/ 79.9	58.8/ 60.3	44.1/ 36.5	64/ 55	50.9/ 50.5	56.1/ 54.4	49.7/ 43.8	60.1/ 63.7
**Dyslipidaemia (%)**	75/ 80	86.2/ 86.3	82/ 81.1	42.4/38.4	63.8/ 57.3	63/ 64	37.6/ 38.3	41.1/ 42.8	41.7/ 45.5	50.6/ 54.7
**ACS at presentation (%)**	23/ 25	26.9/ 24.5	9.8/ 16.4	72.3/ 75.9	100/ 100 (only STEMI)	34/ 37	53.6/ 54.6	100/100	100/100 (only STEMI)	59.1/ NA
**Previous MI (%)**	28.0/ 29.0	21.5/ 22.0	16/ 23.9	16.8/ 16.0	2.1/ 3.1	18/ 14	18/ 18.7	NA	3.5/ 3.5	17.2/ 18.1
**Previous PCI (%)**	12.0/ 9.0	NA	3.4/ 5.2	9.7/8.0	4.2/ 3.1	31/ 32	21.9/ 20.0	NA	3.4/ 3.8	9.4/ 15.2
**DAPT per protocol**	At least 6 months	At least 1 year	At least 1 year	At least 1 year	At least 1 year	At least 6 months	At least 1 year	1 year	1 year	1 year
**On DAPT at 2 yrs. (%)**	36.2/ 34.3	66/ 65.6	52.3/ 50.7	NA	NA	21/ 15	17.5/ 15.6	NA	5.8/ 17.0	5.7/ NA
**Lesions**										
**Randomized, n**	364/ 182	1385/ 713	275/ 137	251/252	95/ 98	112/ 96	1237/ 1209	NA	NA	355/ NA
**ACC/ AHA B2/C (%)**	46/ 49	68.7/ 72.5	76/ 75.9	74.9/ 72.1	NA	35/ 29	55.0/ 51.0	48/42 (C)	NA	38.1/ NA
**Calcification** **(moderate/ severe, %)**	13/ 15.5	NA	34.6/ 43.7	17.5/15.5	NA	NA	30.0/ 28.0	NA	NA	42.2/ NA
**Bifurcation (%)**	0/ 0	0/ 0	0/ 0	50.2/ 48.6	NA	NA	5.0/6.0	NA	NA	21.3/ NA
**Lesion length (mm)**	13.8/ 13.8	12.6/ 13.1	13.5/ 13.3	14.1/ 13.9	12.88/ 13.41	NA	19.1/ 18.8	NA	NA	22.10/ NA
**Pre-procedural RVD (mm)**	2.6/ 2.6	2.67/ 2.65	2.72/ 2.79	2.81/ 2.82	2.86/ 2.76	2.77/ 2.39	2.67/ NA	NA	NA	2.42/ NA
**Pre-procedural DS (%)**	59/ 60	65.3/ 65.9	64.6/ 64.7	65.3/ 64.5	89.5/ 89.9	NA	NA	NA	NA	59.13/ NA
**Pre-dilatation (%)**	100/ 99	100/ 100	100/ 100	99.6/ 98.0	55.8/ 51.0	97/ 86	97.0/ 91.0	NA	81.0/ 29.0	89.8/ NA
**Intravascular imaging (%)**	100/ 100	11.2/ 10.8	68.8/ 68.7	0.4/ 0.4	NA	NA	NA	23/ NA	NA	39.0/ NA
**Post-dilatation (%)**	61/ 59	65.5/ 51.2	82.2/ 77.4	63.0/ 54.4	50.5/ 25.5	31/ 34	74.0/ 49.0	55.2/ NA	36.3/ 15.2	53.3/ NA
**Maximum pressure (atm)**	14.2/ 15.0	15.4/ 15.4	14.7/ 15.1	16.8/ 16.9	15.8/ 18.6	13.6/ 14.6	15.4/ 15.6	20/ NA	NA/ NA	15.5/ NA
**In-device MLD (mm)**	2.22/ 2.50	2.37/ 2.49	2.42/ 2.64	2.48/ 2.59	2.46/ 2.46	2.56/ 2.62	NA	NA	NA	2.30/ NA
**Post-procedural DS (%)**	16/ 10	11.6/ 6.4	11.8/ 7.1	12.2/ 8.7	14.1/ 13.4	9.3/ 8.1	17.0/ NR	NA	NA	16.90/ NA

Values are presented as means or percentages and are described as BVS/ DES. ACS: acute coronary syndrome; DAPT: dual antiplatelet therapy; DS: diameter stenosis; MLD: minimum lumen diameter; NA: not available; RVD: reference vessel diameter.

### Clinical outcomes

**Fig 2:**
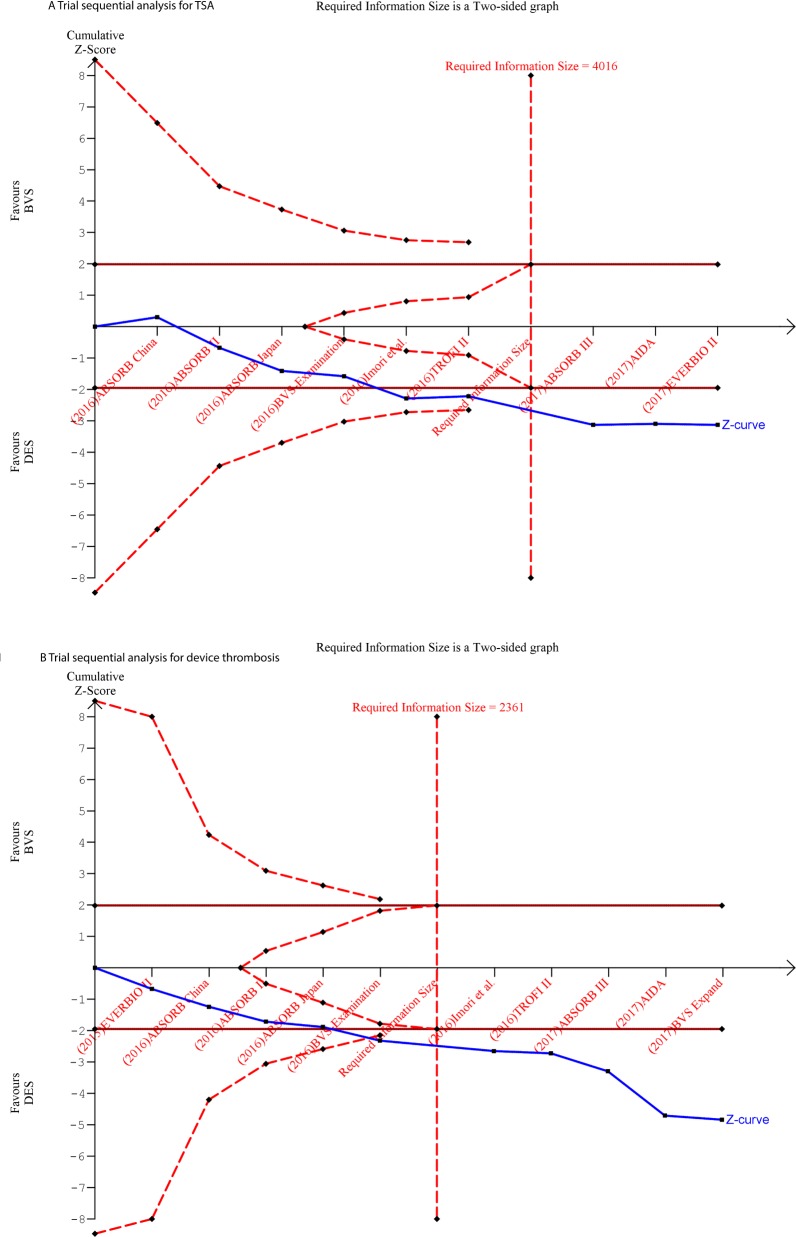
2A and 2B. Trial Sequential Analysis for primary endpoint Target Lesion Failure (A) and secondary endpoint definite/probable device thrombosis (B). The red dotted line represents the trial sequential monitoring boundaries and the futility boundaries. The solid dark red line illustrates the conventional level of significance (p = 0.05). The cumulative Z score (solid blue line) crosses both the conventional boundary and the trial sequential monitoring boundary, indicating sufficient and conclusive evidence.

**Fig 3:**
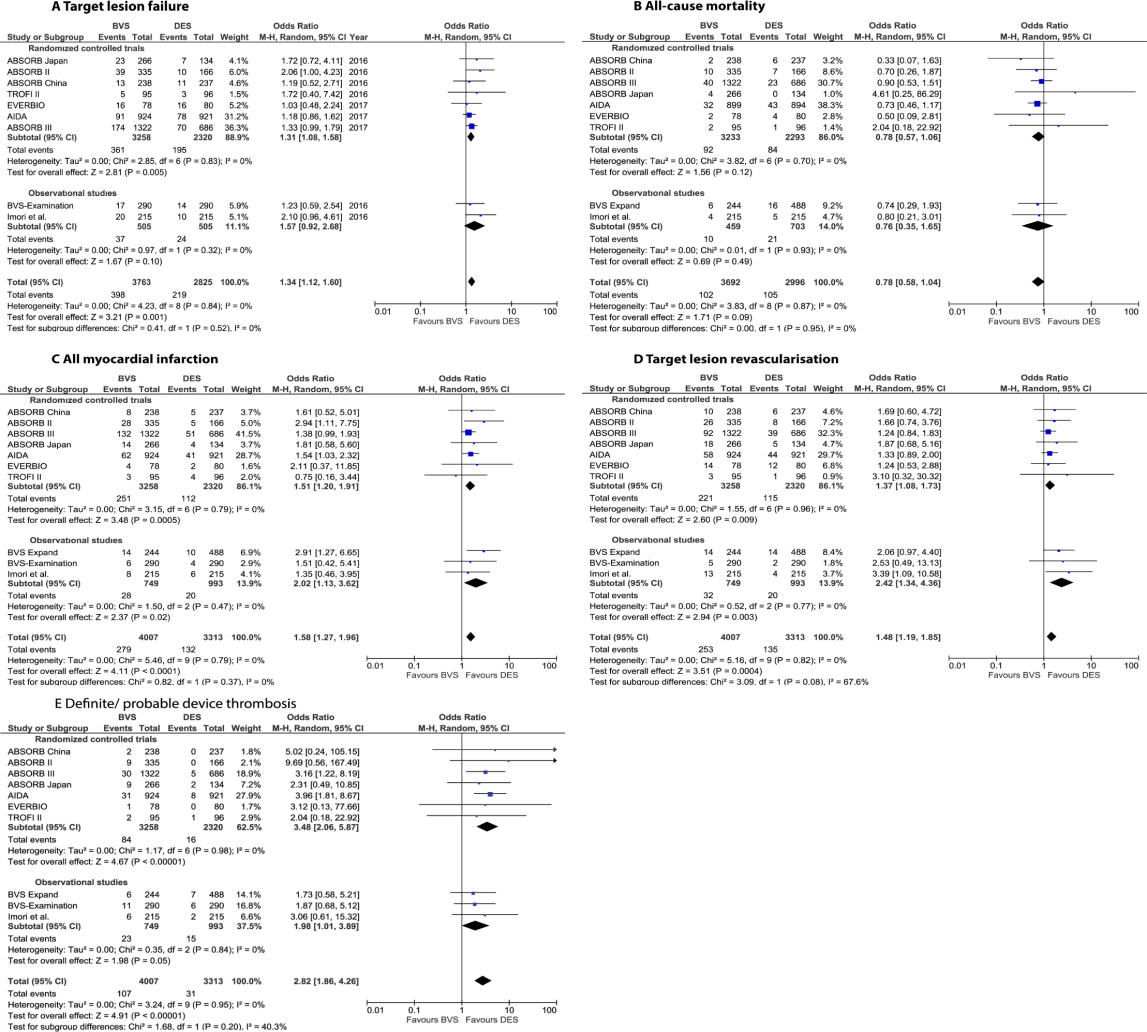
3A – 3E. Forest plots (random effects models) for primary and secondary endpoint of bioresorbable vascular scaffolds versus drug-eluting stents. (A) Target lesion failure, (B) All-cause mortality, (C) All myocardial infarction, (D) Target lesion revascularization. RCTs reported ischemia-driven TLR and observational studies reported all TLR. (E) Definite/ probable device thrombosis. CI: confidence interval; M-H: Mantel-Haenszel; OR: odds ratio.

In the TSA for the primary endpoint, the cumulative Z-curve did cross the TSA monitoring boundary, indicating that there were a sufficient number of patients to consider this a valid analysis (Fig 2A). All studies but one (BVS Expand) reported on TLF. Overall, TLF occurred in 617 patients during the mid-term follow-up, with a significantly higher risk in BVS-treated patients (OR 1.34 [95% CI: 1.12–1.60], p = 0.001 and I^2^ = 0%) (Fig 3A). A subanalysis of RCTs showed only a significantly similar increased OR (1.31 [95% CI: 1.08–1.58], p = 0.005 and I^2^ = 0%). The pooled OR across the observational studies was numerically higher, but with a larger 95% CI (OR 1.57 [95% CI: 0.92–2.68, p = 0.10, I^2^ = 0%).

See S2 and S8 Figs for the sensitivity analyses and S3–S7 Figs for fixed effects models of the primary and secondary endpoints.

Secondary endpoints. All-cause mortality occurred in 207 patients, without a statistically significant difference between both patient groups (OR 0.78 [95% CI: 0.56–1.37], p = 0.09, I^2^ = 0%). Results for the pooled RCT and pooled observational study subgroups were similar (Fig 3B).

The risks of myocardial infarction and TLR were significantly increased for BVS compared with DES (Fig 3C and 3D). Finally, patients with BVS had a higher risk for definite or probable device thrombosis, with ORs of 2.82 (95% CI: 1.86–3.89], p<0.001 and I^2^ = 40.3%), 3.48 (95% CI: 2.06–5.87, p<0.001 and I^2^ = 0%) and 2.82 (95% CI: 1.86–4.26, p<0.001 and I^2^ = 0%), respectively, for the total cohort, RCTs only and observational data only (Fig 3E).

### Landmark analysis

Table 3 summarizes event rates and ORs in the periods up to 1 year, 1–2 years and 2–3 years (for those studies that reported 1- and 2-year and 3-year results of the outcomes of interest: ABSORB II, ABSORB Japan, ABSORB China, ABSORB III). In the first year, the risks of myocardial infarction and device thrombosis were significantly increased in BVS patients. During the second year, all event rates for both BVS and DES were lower, but the increased risk for BVS remained. The OR for late device thrombosis was quadrupled in BVS-treated patients. In the third year, events rates remained lower and no significant differences between the 2 groups existed anymore. However, the OR for device thrombosis in BVS patients continued to be high.

**Table 3:** Outcomes of interest at 0–1 year, 1–2 years and 2–3 years (for included studies that presented outcomes at these time points*).

**Outcome**	Up to 1 year				1 up to 2 years				2 up to 3 years			
	BVS	DES	OR (95% CI)	P	BVS	DES	OR (95% CI)	P	BVS	DES	OR (95% CI)	P
**TLF (%)**	6.39	5.15	1.24 (0.97–1.58)	0.09	4.43	2.55	1.55 (0.98–2.46)	0.06	1.20	0.34	2.75 (0.97–7.78)	0.06
**All-cause mortality (%)**	1.17	1.49	0.90 (0.33–2.43)	0.83	1.10	1.73	0.65 (0.4–1.05)	0.08	0.20	1.88	0.14 (0.01–1.46)	0.10
**Myocardial infarction (%)**	5.15	3.50	1.38 (1.04–1.83)	0.03	2.20	1.01	2.17 (1.30–3.62)	0.003	1.36	0.94	1.18 (0.59–2.37)	0.64
**ID-TLR (%)**	3.08	2.57	1.26 (0.90–1.77)	0.18	2.87	1.59	1.67 (0.97–2.87)	0.06	2.11	1.02	1.79 (0.62–5.15)	0.28
**Def/ prob device thrombosis (%)**	1.60	0.61	2.45 (1.35–4.46)	0.03	0.86	0.10	4.75 (1.63–13.82)	0.004	0.53	0.00	3.79 (0.67–21.37)	0.13

*ABSORB II, ABSORB III, ABSORB China, ABSORB Japan. Def/ prob: definite/probable; OR: Odds ratio; ID-TLR: ischemia driven target lesion revascularization; TLF: target lesion failure

### Definite/Probable device thrombosis

For the secondary endpoint definite or probable device thrombosis, we specifically investigated early (0–30 days), late (31 days-1 year) and very late (> 1 year) device thrombosis (for studies that reported the outcome of interest at these three time points). Event rates for early thrombosis were 1.07% for BVS versus 0.51% for DES. This resulted in an increased risk for BVS (OR 1.96 [95% CI: 1.01–3.81], p = 0.05). Late device thrombosis event rates were 0.53% for BVS versus 0.09% for DES (OR 3.14 [95% CI: 0.83–11.82, p = 0.09). Rates of very late device thrombosis up to three years were 1.09% for BVS compared to 0.0% for DES (OR 6.10 [95% CI: 1.40–26.65], p = 0.02).

The sensitivity analysis results can be found in S2 Fig.

### Quality assessment

Quality assessments for both RCTs and observational studies are provided in the S2 and S3 Tables. All RCTs had a low risk of bias, while the observational studies had a low/low-moderate risk of bias (all scored 7 out of 9). To assess a possible publication bias, a funnel plot for TLF was derived (S1 Fig).

## Discussion

This study included 7320 patients, to report on the mid-term clinical outcomes of the Absorb BVS compared with second-generation DES. Compared to other meta-analyses [11–14], our analysis included the RCTs and complemented only with propensity matched registries to include the highest quality data available for more complex patients. Using this strategy we were able to perform a sub analysis for RCT and propensity match series representing the more complex none RCT patients and a separate analysis for 2 to 3 year outcomes. Furthermore, a trial sequential; analysis was performed and also, several sensitivity analyses were done such an analysis of more complex patients versus non-complex patients.

The main findings of this meta-analysis are: 1) BVS-treated patients were at higher risk for TLF, MI, TLR and device thrombosis compared with second-generation DES, across all studies included in this meta-analysis; 2) this did not result in an increased risk of all-cause mortality; 3) based on studies that have reported clinical outcomes of interest at 1, 2 and 3 years of follow-up, risks of TLF, MI, TLR and especially the risk of very late device thrombosis, continued to be higher for BVS in following years after device implantation.

In our study, propensity matched registries were included. There are some advantages of registries over clinical trials. Firstly, registries handle less strict in- and exclusion criteria and therefore create a more ‘real-world’ patient population [15]. Results originating from registries are better generalizable. Secondly, registries often make use of longer-term follow-up then duration of follow-up observed in RCTs. Thirdly, the larger amount of events makes the identification of rare events, such as ScT, possible. Fourth, as registries integrate data less selected patients, receiving care in diverse clinical settings, they are able to better investigate specific subgroups that are often underrepresented in clinical trials.

Initial study designs for BVS, based on the concept of temporary vascular support, hypothesized non-inferiority at one year and a reduction in TLF of approximately 50% beyond the first year. In this analysis, we demonstrated that event rates were highest during the first year after PCI and, for all endpoints except all-cause mortality; the use of BVS was associated with significantly higher risks of events. The mid-term results in this meta-analysis are in line with previous results [12, 16–21]. Beyond 1 year, event rates were lower than during the first year, but outcomes such as device thrombosis, myocardial infarction and the primary endpoint–TLF–remained not in favour of BVS.

Four RCT’s reported their three-year results and one RCT presented four-year results. All revealed continued higher event rates for BVS. During the EuroPCR 2017 congress, longer term data of several large single-arm registries, that included higher percentages of complex patients, was presented and with varying results [22].

### Definite/Probable device thrombosis

In our study, we demonstrated that the risk of definite device thrombosis was almost three times higher for BVS. Meta-analyses investigating device thrombosis in BVS compared with DES have reported an increased risk of device thrombosis for BVS [5, 23, 24]. Multiple factors have been reported to be associated with scaffold thrombosis, such as a suboptimal implantation strategy, overlap, ostial lesions and decreased left ventricular ejection fraction [25]. Moreover, the first-generation BVS has a strut thickness considerably larger than the competitor metallic DES and similar to first-generation metallic DES. Scaffold thrombosis might be triggered by the smaller minimum lumen diameter and minimum lumen area at the end of the procedure, as previously demonstrated [26]. This has the most impact on smaller vessels (with a diameter <2.5 mm visual or 2.25 mm by quantitative coronary analysis (QCA).

Early device thrombosis is generally considered to be procedure-related, when the characteristics of the device and operators experience are important factors.

The resorption process of the BVS might influence the mechanisms for very late scaffold thrombosis. It has been postulated that the disintegration of uncovered and malapposed struts (due to resorption-related scaffold discontinuity) might trigger the inflammatory process and thrombus formation, potentially for up to 3 years (18, 26, 27).

### Recent setback

Recently, the ABSORB BVS suffered a setback after the 3-year results of the ABSORB II trial demonstrated similar vasomotion between BVS and everolimus-eluting DES and a greater late lumen loss for BVS. [27, 28] The FDA came with a safety alert after the 2-year results of the largest RCT, the ABSORB III, were presented during the ACC congress in March 2017. The AIDA trial even published their 2-year results earlier than expected after the safety monitoring board recommended to release the preliminary data due to safety concerns (hazard ratio of 3.87 for device thrombosis at 2 years; 95% CI: 1.78–8.42; p = <0.001). As a consequence, the current generation BVS has been taken out of the market. Just recently, a Task Force of ESC and EAPCI stated that bioresorbable scaffolds should not be preferred above the current used metallic DES [29]. These unfavourable findings were again confirmed during the 2017 TCT congress in Denver, USA on October the 31^th^. [30–32]

### Possible solutions and future outlook

It remains uncertain whether implantation technique could improve outcomes. The basic concept of optimal implantation includes proper lesion preparation, adequate sizing (avoiding small vessels <2.5 mm) and high-pressure post-dilatation, also known as PSP. In retrospective analyses, this implantation strategy showed a reduction in TLF [25] [22, 33–35]. Also, the 30-day ABSORB IV results revealed lower device thrombosis rates, when implantation of stents/ scaffolds in small vessels was minimalized. [36] The prospective study ‘IT-DIAPPEARS’ showed that when a predefined implantation technique was performed, one-year outcomes were favourable with a def/ prob ScT rate of 0.9%. [37] However, our meta-analysis was not able to correctly assess the influence of PSP on procedural and clinical outcomes, as the included studies did not apply high rates of dedicated implantation strategy.

Furthermore, whether DAPT prolongation could prevent late occurrence of scaffold thrombosis was to be investigated. DAPT termination is a risk factor for device thrombosis, and a possible relationship between scaffold thrombosis and DAPT termination has been described. However, information on the precise duration of DAPT after BVS implantation is lacking and, up to this moment, no dedicated studies exist on this important issue. A recently published review has suggested several considerations for DAPT duration in BVS patients [38]. In metal stents, prolongation of DAPT up to 30 months showed to reduce thrombotic events [39]. The new generation device should have thinner struts, better mechanical properties and shorter resorption time to facilitate easy implantation strategies and to prevent intraluminal dismantling [40].

### Limitations

The most important limitation is the use of unpublished data in the form of meeting presentations. Secondly, the meta-analysis was performed using study-level data rather than patient-level data, so time-to-event curves were not possible. Thirdly, heterogeneity existed in baseline characteristics of included patients and also in protocols, study designs and definitions across the studies. Furthermore, the patients included in the RCTs (which provided most patients) were highly selected (except for AIDA) and, therefore, extrapolation to the real world is difficult. Besides, we were not able to completely exclude potential confounders in the observational registries. However these studies were based on propensity matching. Fourthly, the large AIDA RCT had a median follow-up duration of 1.93 years (range 1−3.3 years); thus this trial did not report outcomes at exactly 2 years.

Longer follow-up will be necessary to get a better view of the low-frequency endpoint mortality.

To assess possible publication bias, we provided a funnel plot in S1 Fig. However, this plot should be interpreted with caution as we included ten studies. There was also a lack of important information on DAPT status (duration of DAPT, reasons for interruption or early termination, type of P2Y_12_ inhibitor). Lastly, the current data only apply for the Absorb BVS and not for other bioresorbable devices.

## Conclusions

At mid-term follow-up, patients treated with Absorb BVS showed a higher risk of TLF, myocardial infarction, TLR and definite or probable device thrombosis. Beyond 1 year, it was mainly the risk of late device thrombosis that was increased. However, this did not result in a higher risk of all-cause mortality. Despite these unfavourable mid-term outcomes, long-term follow-up will be necessary to investigate any potential late benefits of BVS over DES as this device was not able to show any clinical benefit up to 3 years. Specific registries and post-hoc analyses of larger RCTs identified potential improvements in patient and lesion selection. A device specific implantation strategy is another factor that can result in better outcomes. As long as this has not been demonstrated in prospective and dedicated studies such as ABSORB III (NCT01751906), ABSORB IV (NCT02173379) and Compare Absorb (NCT02486068) operators should not use this version in routine practice.

## Supporting information

S1 Fig Funnel plot for the primary endpoint. (DOCX)

S2 Fig Sensitivity analysis for TLF and device thrombosis. **Non-Complex Studies Versus Complex Studies.** Random effects effects model. CI: confidence interval; M-H: Mantel-Haenszel; OR: odds ratio.
(DOCX)

S3 Fig Target lesion failure. Fixed effects model. CI: confidence interval; M-H: Mantel-Haenszel; OR: odds ratio.
(DOCX)

S4 Fig All-cause mortality. Fixed effects model. CI: confidence interval; M-H: Mantel-Haenszel; OR: odds ratio.
(DOCX)

S5 Fig Myocardial infarction. Fixed effects model. CI: confidence interval; M-H: Mantel-Haenszel; OR: odds ratio.
(DOCX)

S6 Fig Target lesion revascularization. Fixed effects model. CI: confidence interval; M-H: Mantel-Haenszel; OR: odds ratio.
(DOCX)

S7 Fig Definite/Probable device thrombosis. Fixed effects model. CI: confidence interval; M-H: Mantel-Haenszel; OR: odds ratio.
(DOCX)

S8 Fig Sensitivity analysis for TLF. RCTs versus propensity matched studies. Random effects effects model. CI: confidence interval; M-H: Mantel-Haenszel; OR: odds ratio.
(DOCX)

S1 Text Literature search in the most important online databases. (DOCX)

S1 Table Definitions of clinical outcomes per study. CABG: coronary artery bypass grafting; CK: creatine kinase; CK-MB: creatine kinase myoglobulin; ID-TLR: ischemia-driven target lesion revascularization; MI: myocardial infarction, ULN: upper limit of normal.
(DOCX)

S2 Table Assessment of risk of bias for randomized controlled trials. CEC: clinical event committee; IWRS: interactive web-based response system.
(DOCX)

S3 Table Quality assessment for observational studies. Score of nine is maximum score (= lowest risk of bias).
(DOCX)

S4 Table Checklist for PRISMA guidelines. (DOCX)

## Abbreviations

BVS: Bioresorbable vascular scaffold
CI: Confidence interval
DAPT: Dual antiplatelet therapy
DES: Drug-eluting stent
OR: Odds ratio
RCT: Randomized controlled trials
STEMI: ST-segment elevation myocardial infarction
TLF: Target lesion failure
TLR: Target lesion revascularization
TSA: Trial sequential analysis
