# Supplementary figures and images for: Mid-term outcomes of the Absorb BVS versus second-generation DES: A systematic review and meta-analysis
Source: PLoS One. 2018 May 9;13(5):e0197119. doi: 10.1371/journal.pone.0197119 (PMC5942828; doi:10.1371/journal.pone.0197119)

**S1 Fig. Funnel plot for TLF**

**
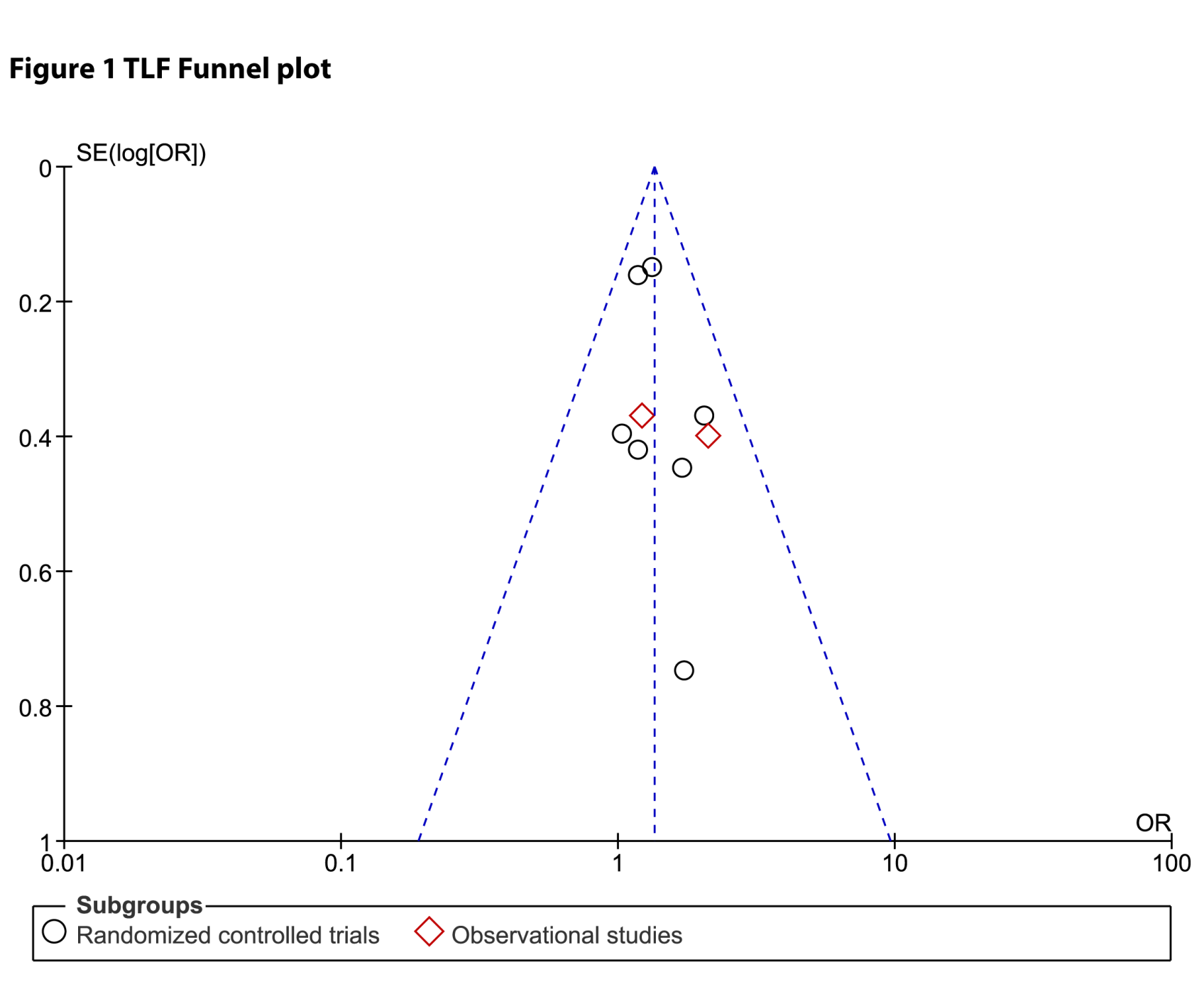
**

Supplement: S1 Fig — (DOCX) [file pone.0197119.s001.docx]

**S3 Fig. Target lesion failure**

**
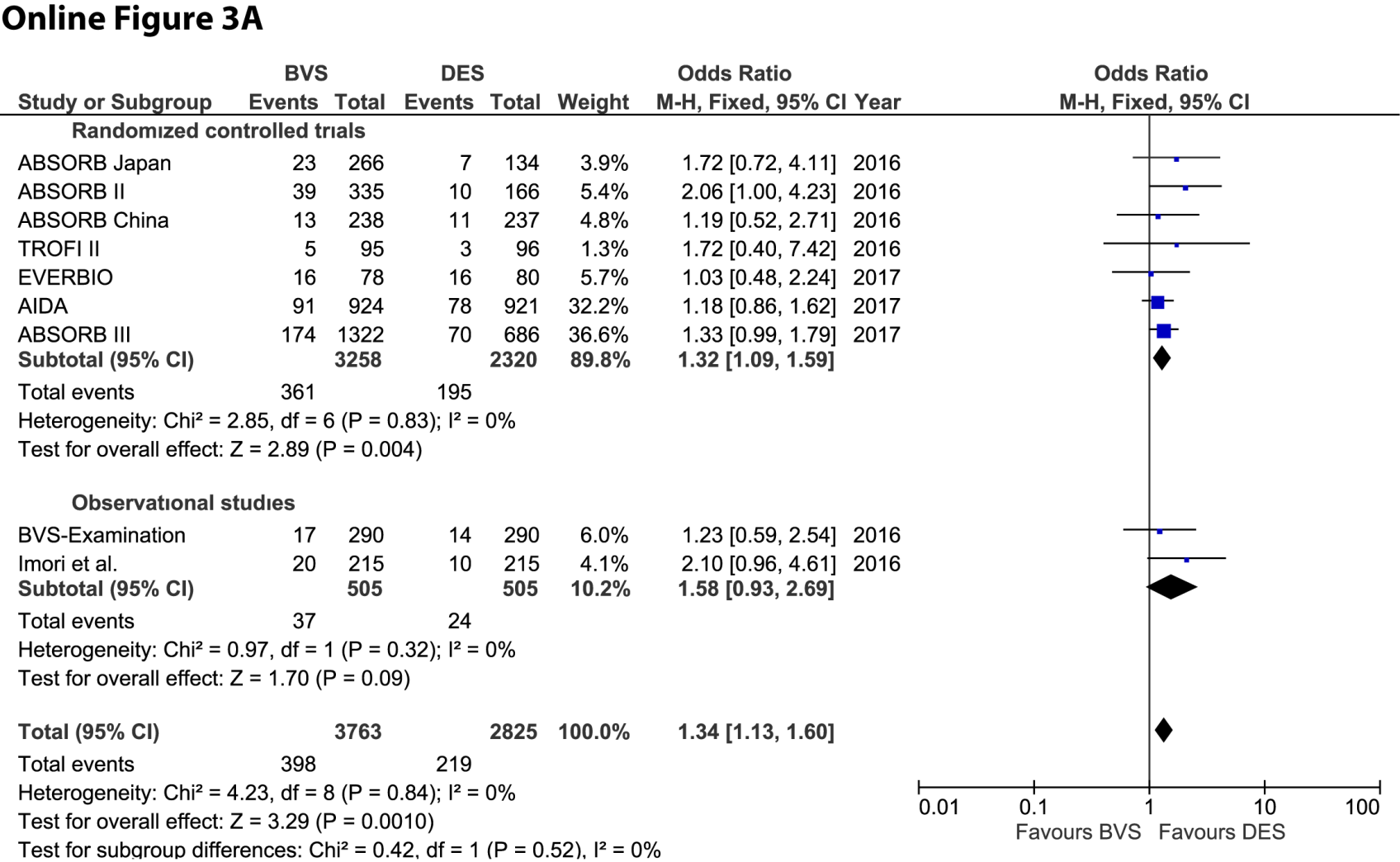
**

Supplement: S3 Fig — Fixed effects model. CI: confidence interval; M-H: Mantel-Haenszel; OR: odds ratio. (DOCX) [file pone.0197119.s003.docx]

**S4 Fig. All-cause mortality**

**
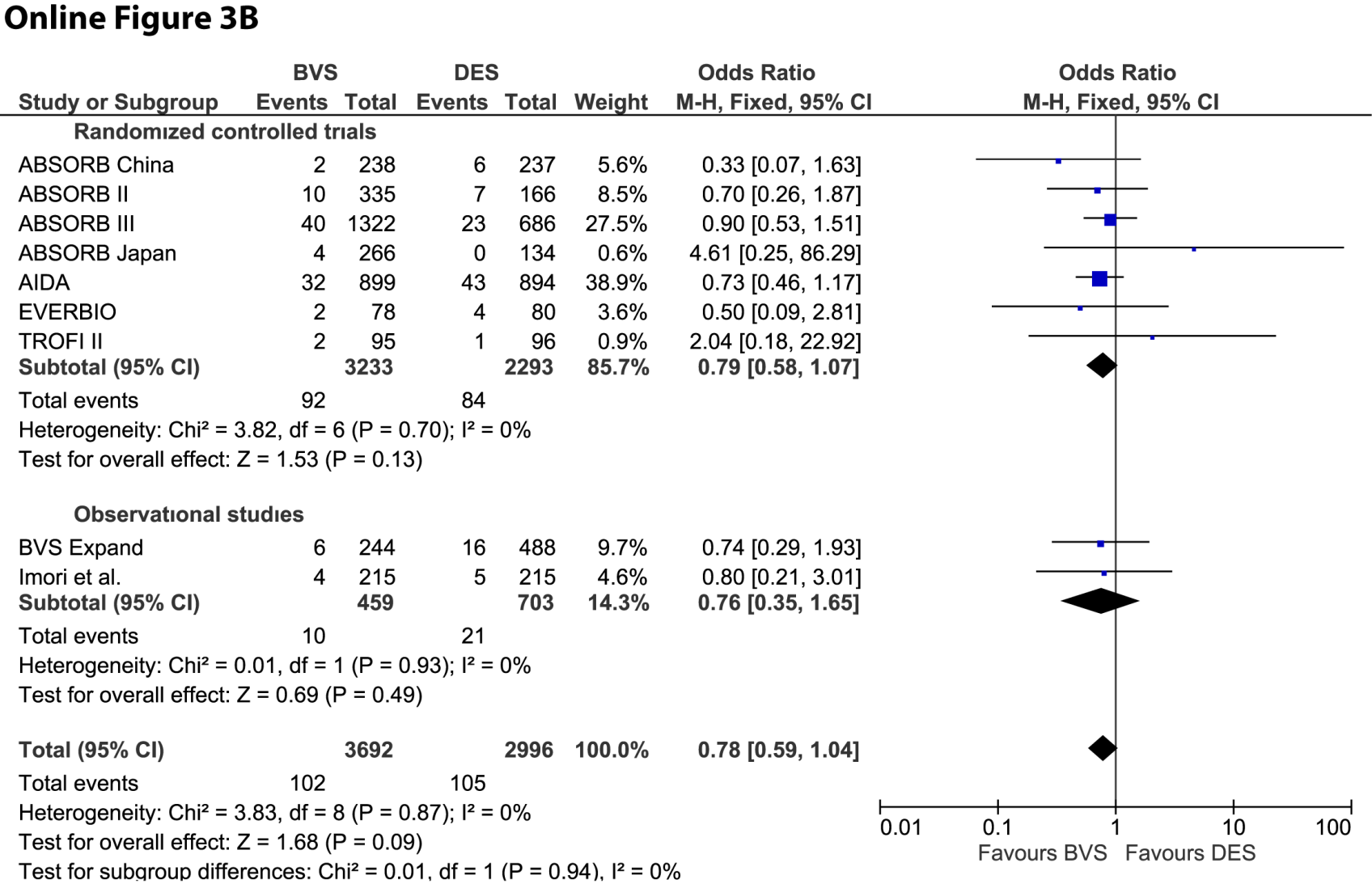
**

Supplement: S4 Fig — Fixed effects model. CI: confidence interval; M-H: Mantel-Haenszel; OR: odds ratio. (DOCX) [file pone.0197119.s004.docx]

**S5 Fig. Myocardial infarction**

**
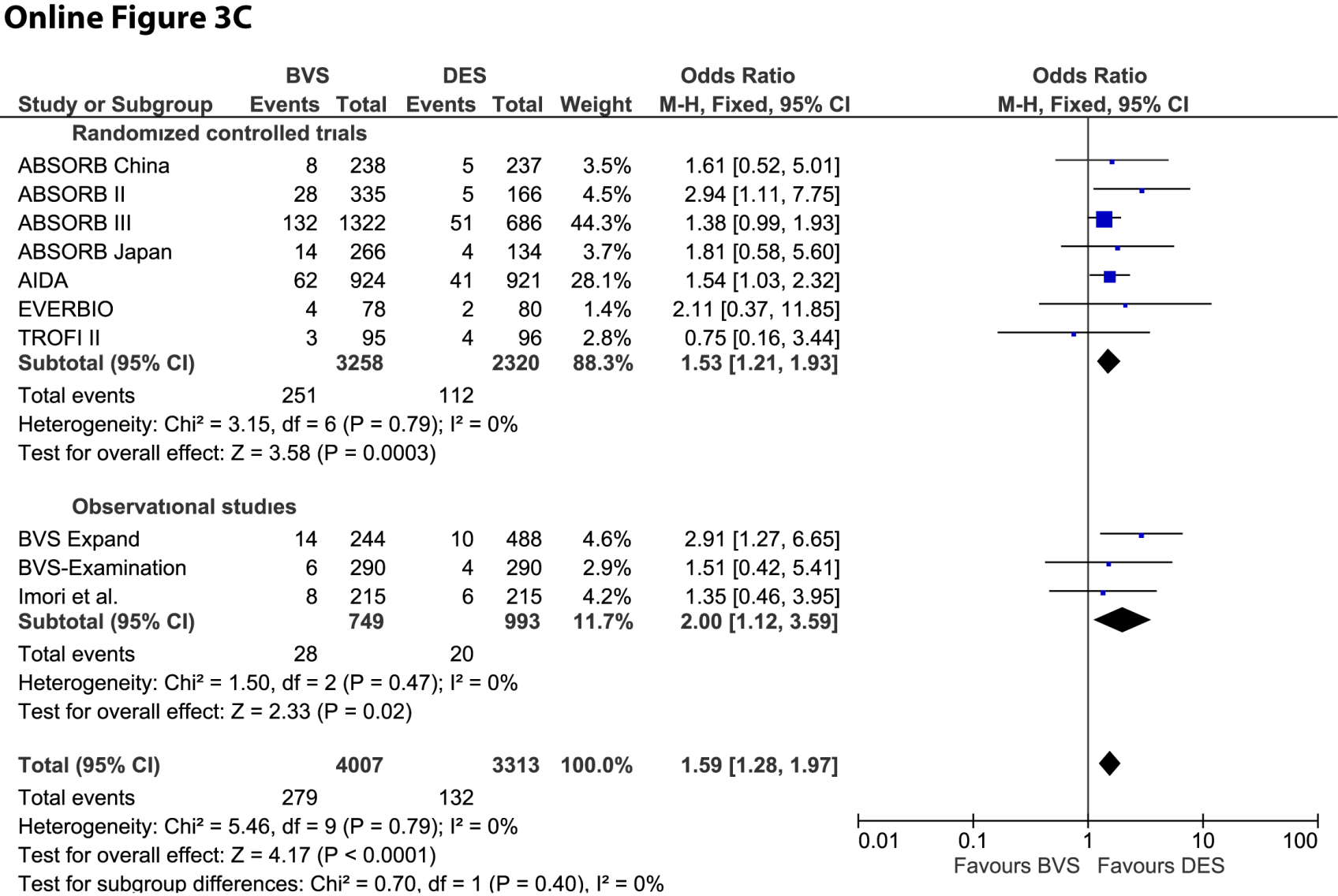
**

Supplement: S5 Fig — Fixed effects model. CI: confidence interval; M-H: Mantel-Haenszel; OR: odds ratio. (DOCX) [file pone.0197119.s005.docx]

**S6 Fig. Target lesion revascularization**

**
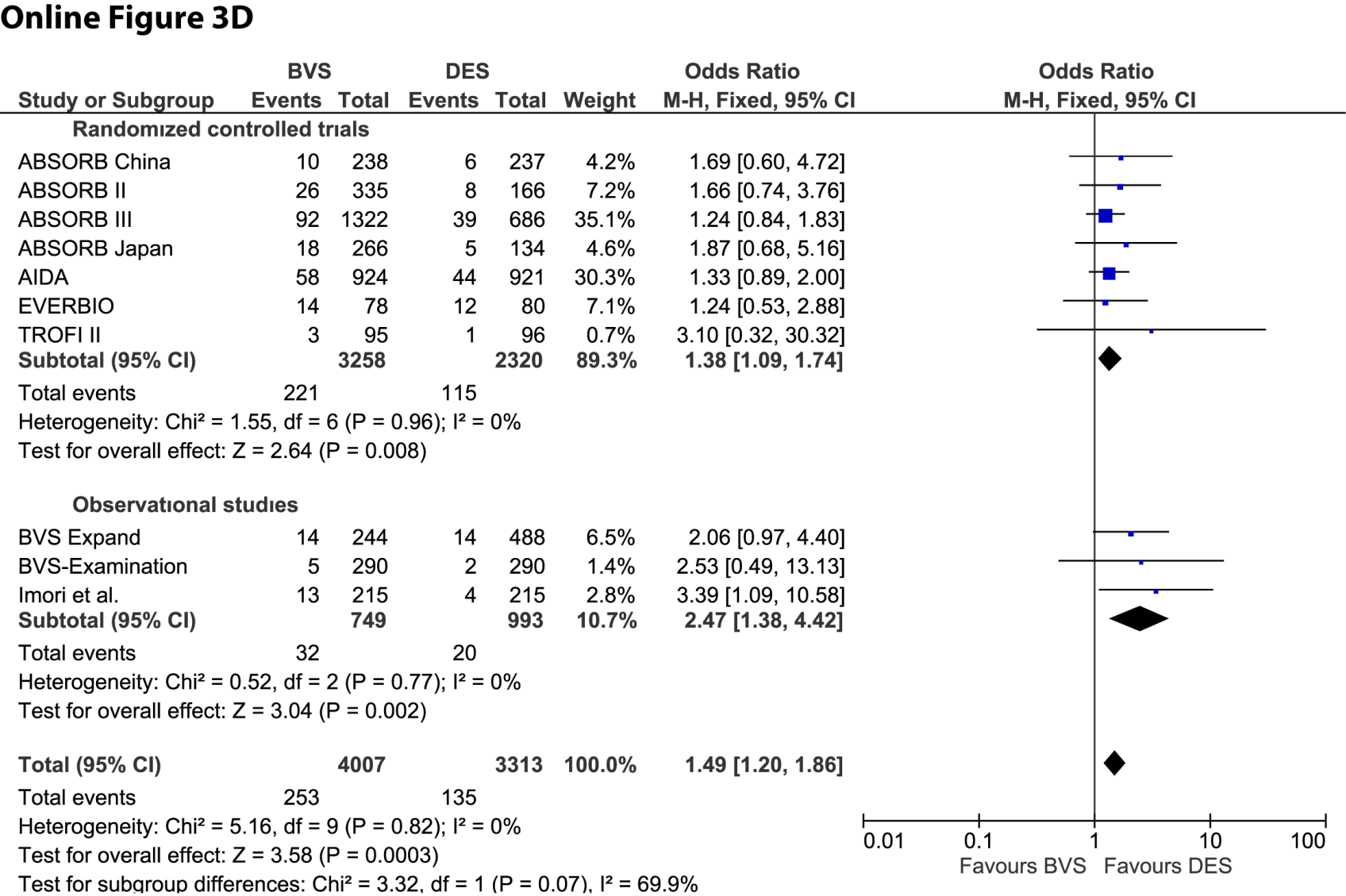
**

Supplement: S6 Fig — Fixed effects model. CI: confidence interval; M-H: Mantel-Haenszel; OR: odds ratio. (DOCX) [file pone.0197119.s006.docx]

**S7 Fig. Definite or probable device thrombosis**

**
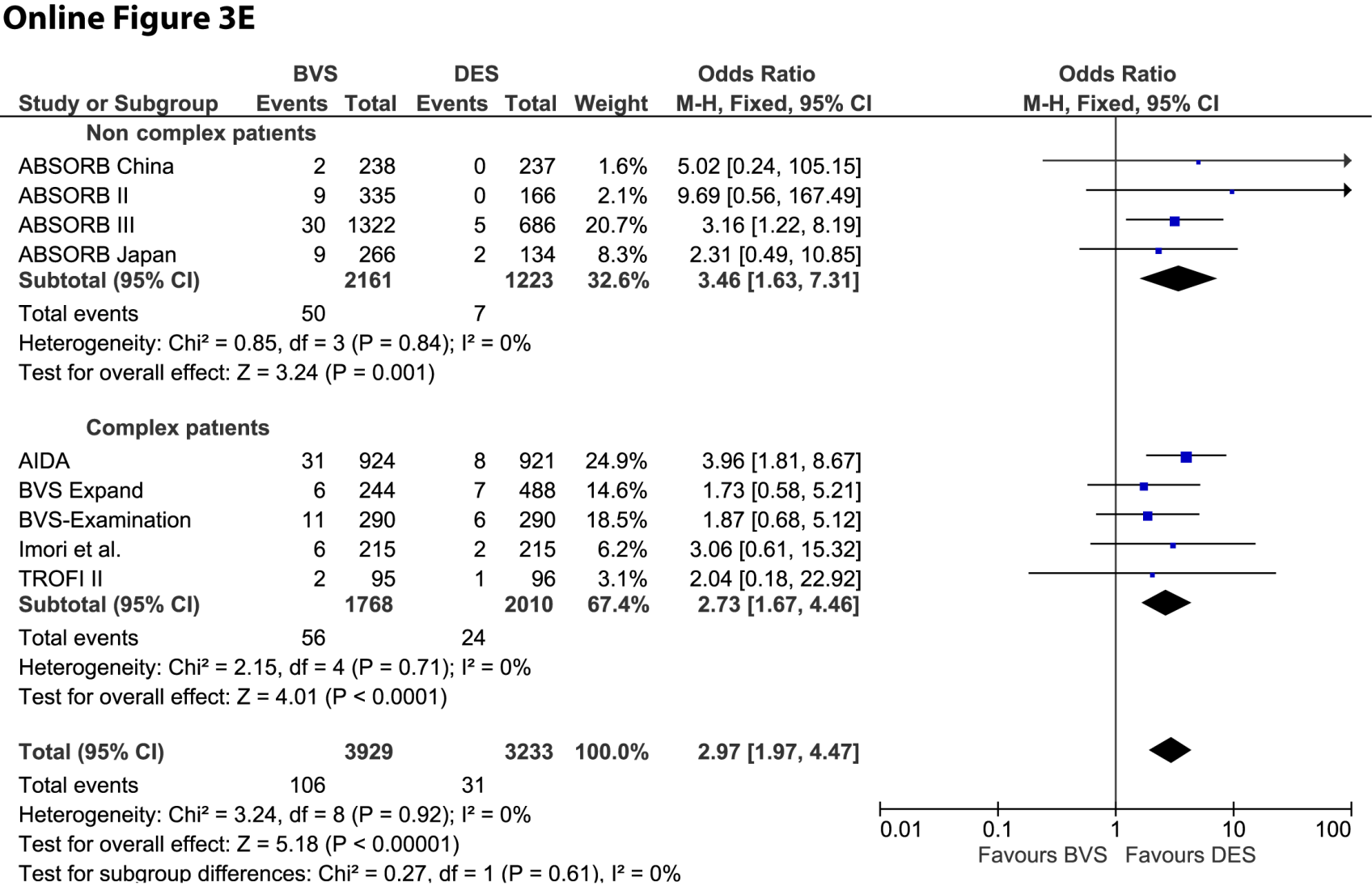
**

Supplement: S7 Fig — Fixed effects model. CI: confidence interval; M-H: Mantel-Haenszel; OR: odds ratio. (DOCX) [file pone.0197119.s007.docx]

**S8 Fig. Sensitivity analysis for TLF. RCTs versus propensity matched studies**

**
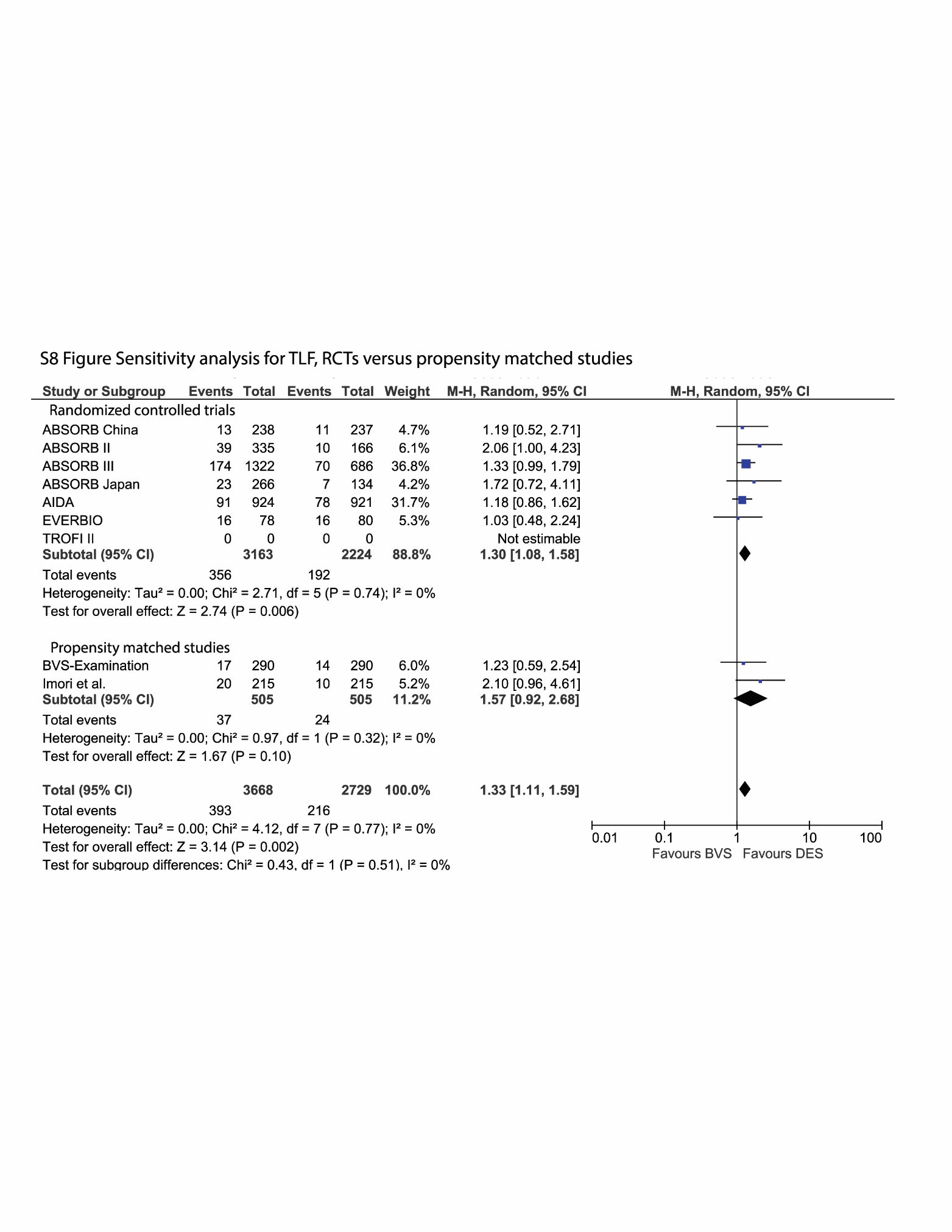
**

Supplement: S8 Fig — Random effects effects model. CI: confidence interval; M-H: Mantel-Haenszel; OR: odds ratio. (DOCX) [file pone.0197119.s008.docx]
