# Supplementary material for: Mid-term outcomes of the Absorb BVS versus second-generation DES: A systematic review and meta-analysis
Source: PLoS One. 2018 May 9;13(5):e0197119. doi: 10.1371/journal.pone.0197119 (PMC5942828; doi:10.1371/journal.pone.0197119)
Supplement: S2 Fig — Non-Complex Studies Versus Complex Studies. Random effects effects model. CI: confidence interval; M-H: Mantel-Haenszel; OR: odds ratio. (DOCX) [file pone.0197119.s002.docx]

**S2 Fig. Sensitivity analysis for TLF and device thrombosis. Non-complex studies versus complex studies**

**
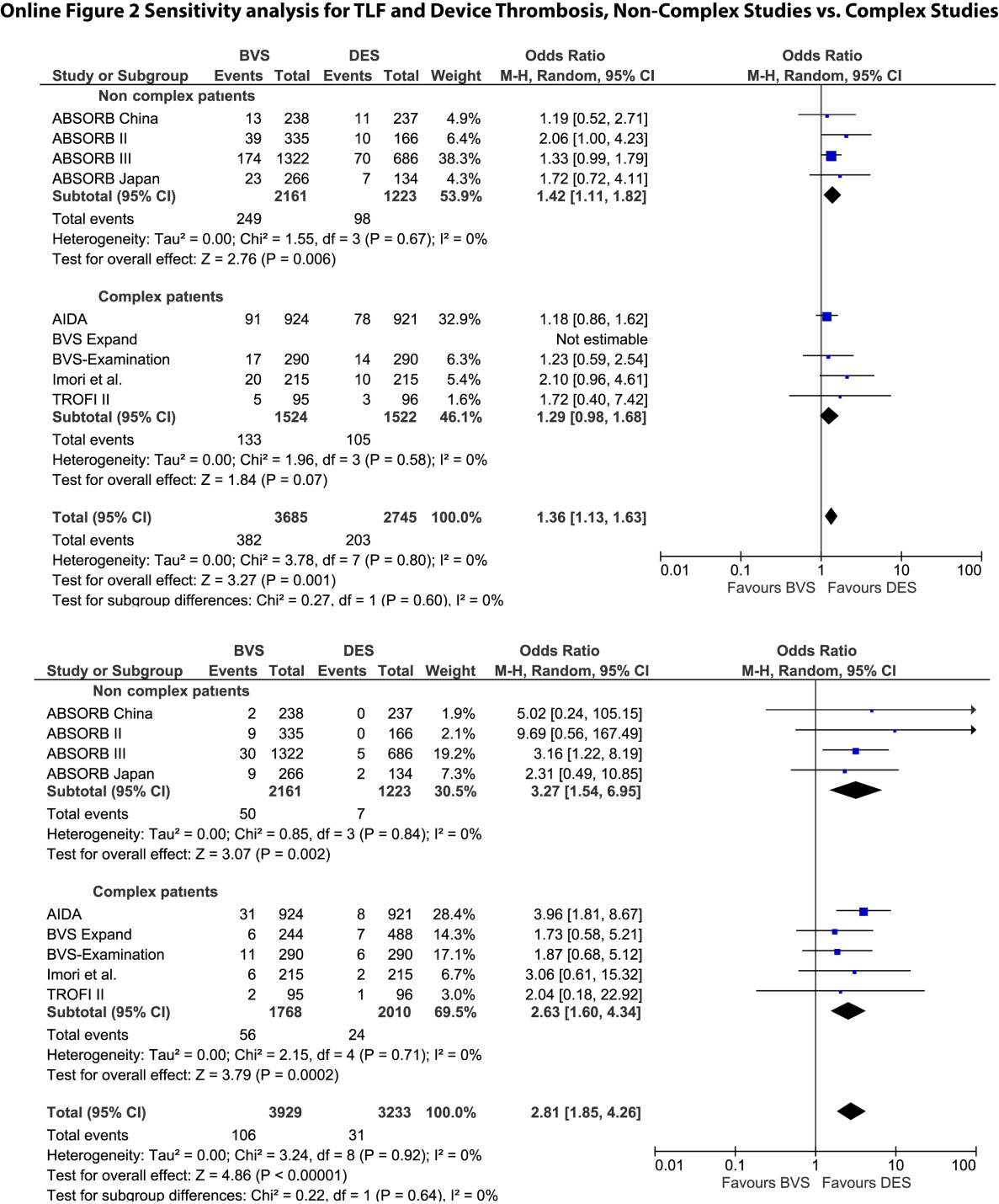
**
