# Supplementary material for: Mid-term outcomes of the Absorb BVS versus second-generation DES: A systematic review and meta-analysis
Source: PLoS One. 2018 May 9;13(5):e0197119. doi: 10.1371/journal.pone.0197119 (PMC5942828; doi:10.1371/journal.pone.0197119)
Supplement: S1 Text — (DOCX) [file pone.0197119.s009.docx]

**S1 Text. Literature searches in most important databases**

**Embase.com**

('bioresorbable vascular stent'/exp OR 'bioresorbable scaffold'/exp OR (((bioresorbab* OR bioabsorbab* OR bvs OR fully-resorbab* ) NEAR/3 (stent* OR scaffold*)) OR (Absorb* NEAR/10 (stent* OR bvs))):ab,ti) AND ('everolimus eluting coronary stent'/exp OR 'drug eluting stent'/mj OR (('drug eluting stent'/de OR 'drug eluting coronary stent'/de OR 'metal stent'/de OR stent/de) AND everolimus/de) OR (((everolimus OR ees ) NEAR/3 (stent* OR des)) OR XIENCE-Xpedition* OR Xience OR ((second-generation OR 2nd-generation OR 2-nd-generation OR non-BVS OR non-bioabsorbable OR non-bioresorbable OR conventional OR current-generation*) NEAR/6 (eluting OR des OR coated*) NEAR/6 stent*)):ab,ti OR 'drug eluting stent*':ti)

**Medline Ovid**

((((bioresorbab* OR bioabsorbab* OR bvs OR fully-resorbab* ) ADJ3 (stent* OR scaffold*)) OR (Absorb* ADJ10 (stent* OR bvs))).ab,ti.) AND (*"Drug-Eluting Stents" OR (("Drug-Eluting Stents"/ OR stents/) AND everolimus/) OR (((everolimus OR ees ) ADJ3 (stent* OR des)) OR XIENCE-Xpedition* OR Xience OR ((second-generation OR 2nd-generation OR 2-nd-generation OR non-BVS OR non-bioabsorbable OR non-bioresorbable OR conventional OR current-generation*) ADJ6 (eluting OR des OR coated*) ADJ6 stent*)).ab,ti. OR "drug eluting stent*".ti.)

**Cochrane**

((((bioresorbab* OR bioabsorbab* OR bvs OR fully-resorbab* ) NEAR/3 (stent* OR scaffold*)) OR (Absorb* NEAR/10 (stent* OR bvs))):ab,ti) AND ((((everolimus OR ees ) NEAR/3 (stent* OR des)) OR XIENCE-Xpedition* OR Xience OR ((second-generation OR 2nd-generation OR 2-nd-generation OR non-BVS OR non-bioabsorbable OR non-bioresorbable OR conventional OR current-generation*) NEAR/6 (eluting OR des OR coated*) NEAR/6 stent*)):ab,ti OR 'drug eluting stent*':ti)
