# Supplementary material for: Mid-term outcomes of the Absorb BVS versus second-generation DES: A systematic review and meta-analysis
Source: PLoS One. 2018 May 9;13(5):e0197119. doi: 10.1371/journal.pone.0197119 (PMC5942828; doi:10.1371/journal.pone.0197119)
Supplement: S1 Table — CABG: coronary artery bypass grafting; CK: creatine kinase; CK-MB: creatine kinase myoglobulin; ID-TLR: ischemia-driven target lesion revascularization; MI: myocardial infarction, ULN: upper limit of normal. (DOCX) [file pone.0197119.s010.docx]

**S1 Table. Definitions of clinical outcomes per study for RCTs and registries**

|  | **ABSORB II** | **ABSORB III** | **ABSORB Japan** | **ABSORB China** | **TROFI II** | **EVERBIO II** | **AIDA** |
| --- | --- | --- | --- | --- | --- | --- | --- |
| **Target lesion failure** | Cardiac death, target-vessel MI, ID-TLR | Cardiac death, target-vessel MI, ID-TLR | Cardiac death, target-vessel MI, ID-TLR | Cardiac death, target-vessel MI, ID-TLR | Cardiac death, MI (not clearly attributable to a non-target vessel), ID-TLR | Cardiac death, target-vessel MI, CD-TLR | Cardiac death, target-vessel MI, CD-TLR |
| **Device thrombosis** | ARC definitions | ARC definitions | ARC definitions | ARC definitions | ARC definitions | ARC definitions | ARC definitions |
| **Myocardial infarction** | New pathological Q-wave or CK rise > 2 of ULN accomplice by CK-MB rise | *Periprocedural:* CK-MB to >5x ULN within 48 hours in cases in which the baseline CK-MB value is <ULN  *Spontaneous:* defined as elevation of troponin >ULN or CK-MB >ULN and ≥ of the following: ischemic symptoms, ischemic ECG changes, development of pathological Q waves, or imaging findings of acute MI | *Periprocedural:*  CK-MB >5x ULN.  *Spontaneous:* Troponin>ULN or CK-MB>ULN and ≥ of the following: ischemic symptoms, ischemic ECG changes, development of pathological Q waves, or imaging findings of acute MI | *Periprocedural:* CK-MB >5× ULN  *Spontaneous:* defined as elevation of troponin >ULN or CK-MB >ULN and ≥ of the following: ischemic symptoms, ischemic ECG changes, development of pathological Q waves, or imaging findings of acute MI | New pathological Q-waves in ≥ 2 contiguous leads (as assessed by the ECG core laboratory) with or without post-procedure troponin, CK or CK-MB levels elevated above normal; Detection of a rise and/or fall of cardiac biomarker values (preferably cardiac troponin) with at least one value above the 99 percentile ULN and ≥ of the following: ischemic symptoms, ischemic ECG changes, development of pathological Q waves, or imaging/ autopsy findings of acute MI | *Periprocedural:* According to Global MI Task Force for the Universal definition of Myocardial Infarction ^35^  *Spontaneous::* Development of new pathological Q waves ≥0.04 s in duration in ≥2 contiguous leads or an elevation of creatine phosphokinase levels to >2 times normal with positive creatine phosphokinase–MB or troponin I levels | ‘Third Universal Myocardial infarction’ definitions^35^ |
| **Target lesion revascularization** | Any clinically indicated repeat PCI of the target lesion or CABG of the target vessel | Any repeat PCI of the target lesion or CABG of the target vessel | Any repeat PCI of the target lesion or CABG of the target vessel | Any repeat PCI of the target lesion or CABG of the target vessel | Any repeat PCI of the target lesion or CABG of the target vessel | Repeat revascularization within the stent or the 5-mm boarders proximal and distal to the stent. | Any repeat PCI of the target lesion or CABG of the target vessel |
| **Death** | All deaths were considered cardiac unless an unequivocal non-cardiac cause was established | All deaths were considered cardiac unless an unequivocal non-cardiac cause was established | All deaths were considered cardiac unless an unequivocal non-cardiac cause was established | All deaths were considered cardiac unless an unequivocal non-cardiac cause was established | All deaths were considered cardiac unless an unequivocal non-cardiac cause was established | All deaths were considered cardiac unless an unequivocal non-cardiac cause was established | All deaths were considered cardiac unless an unequivocal non-cardiac cause was established |

CABG: coronary artery bypass grafting; CK: creatine kinase; CK-MB: creatine kinase myoglobulin; ID-TLR: ischemia-driven target lesion revascularization; MI: myocardial infarction, ULN: upper limit of normal

|  | **BVS-Examination** | **Imori et al.** | **BVS Expand** |
| --- | --- | --- | --- |
| **Target lesion failure** | Cardiac death, target-vessel MI, TLR | NA | Cardiac death, target-vessel MI, ID-TLR |
| **Patient oriented endpoint** | All-cause death, any MI, any revascularization | NA | NA |
| **Device thrombosis** | ARC definitions | ARC definitions | ARC definitions |
| **Myocardial infarction** | Based on Historical Extended Definition of MI (modified ARC Definition according to Vranckx *et al*. ^33^) | NR | Based on Historical Extended Definition of MI (modified ARC Definition according to Vranckx *et al*. ^33^) and per protocol definition of MI also known as the World Health Organization Definition of MI. |

ID-TLR: ischemia-driven target lesion revascularization; MI: myocardial infarction; NA: not applicable; ULN: upper limit of normal
