# Supplementary material for: Mid-term outcomes of the Absorb BVS versus second-generation DES: A systematic review and meta-analysis
Source: PLoS One. 2018 May 9;13(5):e0197119. doi: 10.1371/journal.pone.0197119 (PMC5942828; doi:10.1371/journal.pone.0197119)
Supplement: S2 Table — CEC: clinical event committee; IWRS: interactive web-based response system. (DOCX) [file pone.0197119.s011.docx]

**S2 Table. Assessment of risk of bias for randomized controlled trials**

| Trial | **Random sequence generation** | **Allocation concealment** | **Blinding of participants** | **Blinding of outcome assessment** | **Incomplete outcome data** | **Selective outcome reporting** | **Sample size calculation** | **Sponsor** |
| --- | --- | --- | --- | --- | --- | --- | --- | --- |
| ABSORB II | IWRS | Yes | Yes | Yes (independent CEC) | Yes | No | Yes | Industry |
| ABSORB III | IWRS | Yes | Yes | Yes (independent CEC) | Yes | No | Yes | Industry |
| ABSORB Japan | IWRS | Yes | Yes | Yes (independent CEC) | Yes | No | Yes | Industry |
| ABSORB China | IWRS | Yes | No | Yes (independent CEC) | Yes | No | Yes | Industry |
| AIDA | IWRS | Yes | Yes | Yes (independent CEC) | Yes | No | Yes | Investigator |
| TROFI II | IWRS | Yes | No | Yes (independent CEC) | Yes | No | Yes | Investigator |
| EVERBIO II | IWRS | Yes | Yes | Yes (independent CEC) | Yes | No | Yes | Investigator |

CEC: clinical event committee; IWRS: interactive web-based response system
