# Supplementary material for: Mid-term outcomes of the Absorb BVS versus second-generation DES: A systematic review and meta-analysis
Source: PLoS One. 2018 May 9;13(5):e0197119. doi: 10.1371/journal.pone.0197119 (PMC5942828; doi:10.1371/journal.pone.0197119)
Supplement: S3 Table — Score of nine is maximum score (= lowest risk of bias). (DOCX) [file pone.0197119.s012.docx]

**S3 Table. New Castle-Ottawa scale for case-control studies**

| Study | **Selection** | **Comparability on basis of design and analysis** | **Outcome** |  |
| --- | --- | --- | --- | --- |
| Imori et al. | **** | ***** | ******* |  |
| BVS Examination | **** | ***** | ******* |  |
| BVS Expand | ******** | ***** | ******* |  |

Score of nine is maximum score (= lowest risk of bias)
